# Cerebellar Transcranial Direct Current Stimulation Applied over Multiple Days Does Not Enhance Motor Learning of a Complex Overhand Throwing Task in Young Adults

**DOI:** 10.3390/bioengineering10111265

**Published:** 2023-10-30

**Authors:** Milan Pantovic, Daniel E. Lidstone, Lidio Lima de Albuquerque, Erik W. Wilkins, Irwin A. Munoz, Daniel G. Aynlender, Desiree Morris, Janet S. Dufek, Brach Poston

**Affiliations:** 1 Health and Human Performance Department, Utah Tech University, St. George, UT 84770, USA; milan.pantovic@utahtech.edu; 2 Center for Neurodevelopment and Imaging Research, Kennedy Krieger Institute, Department of Neurology, Johns Hopkins University School of Medicine, Baltimore, MD 21205, USA; lidstone@kennedykrieger.org; 3 School of Health and Applied Human Sciences, University of North Carolina Wilmington, Wilmington, NC 28403, USA; limadeal@uncw.edu; 4 Department of Kinesiology and Nutrition Sciences, University of Nevada Las Vegas, Las Vegas, NV 89154, USA; wilkie1@unlv.nevada.edu (E.W.W.); janet.dufek@unlv.edu (J.S.D.); 5 School of Medicine, University of Nevada-Las Vegas, Las Vegas, NV 89154, USA; munozi2@unlv.nevada.edu (I.A.M.); aynlende@unlv.nevada.edu (D.G.A.); morrid3@unlv.nevada.edu (D.M.)

**Keywords:** motor skill, transcranial magnetic stimulation, transcranial direct current stimulation

## Abstract

Cerebellar transcranial direct current stimulation (tDCS) enhances motor skill and learning in relatively simple motor tasks, but it is unclear if c-tDCS can improve motor performance in complex motor tasks. The purpose of this study was to determine the influence of c-tDCS applied over multiple days on motor learning in a complex overhand throwing task. In a double-blind, randomized, between-subjects, SHAM-controlled, experimental design, 30 young adults were assigned to either a c-tDCS or a SHAM group. Participants completed three identical experiments on consecutive days that involved overhand throwing in a pre-test block, five practice blocks with concurrent c-tDCS, and a post-test block. Overhand throwing endpoint accuracy was quantified as the endpoint error. The first dorsal interosseous muscle motor evoked potential (MEP) amplitude elicited by transcranial magnetic stimulation was used to quantify primary motor cortex (M1) excitability modulations via c-tDCS. Endpoint error significantly decreased over the 3 days of practice, but the magnitude of decrease was not significantly different between the c-tDCS and SHAM group. Similarly, MEP amplitude slightly increased from the pre-tests to the post-tests, but these increases did not differ between groups. These results indicate that multi-day c-tDCS does not improve motor learning in an overhand throwing task or increase M1 excitability.

## 1. Introduction

Transcranial direct current stimulation (tDCS) delivered to the cerebellum (c-tDCS) has shown the ability to induce acute enhancements in skill acquisition in a variety of motor tasks [1,2,3,4,5]. Specifically, most studies have observed motor skill increases on the order of 10–15% when c-tDCS is applied before or during motor practice compared with task practice alone [6,7,8,9,10,11]. These improvements can approach the results obtained when tDCS is applied to the primary motor cortex (M1) [8,12]. M1 has been the brain area most commonly targeted by tDCS and stimulation of this area has generally been found to confer the greatest performance benefits [1,13,14,15,16]. However, c-tDCS may be able to elicit similar or greater effects when compared with M1-tDCS in specific experimental conditions such as adaptation-learning paradigms [4,8]. In addition, c-tDCS may be more effective for specific motor tasks where execution is highly dependent on the specialized contributions of the cerebellum in motor control [2,3,4,5].

The vast majority of motor skill studies that have applied tDCS to any brain area have involved a single stimulation session, usually lasting between 10 and 25 min. However, a few studies [13,14] that applied M1-tDCS for 3–5 consecutive days reported cumulative effects that produced 20–40% enhancements in total motor learning compared to SHAM stimulation in a sequential visual isometric pinch force task (SVIPT) of the thumb and index fingers. In addition, Cantarero et al. (2015) [12] delivered c-tDCS over 3 consecutive days simultaneous with the same SVIPT and found substantial increases in motor learning in an anodal c-tDCS group compared to both a cathodal c-tDCS and a SHAM stimulation group. Interestingly, the phase of learning in which the gains in motor performance were predominantly realized differed between M1-tDCS and c-tDCS in these studies. M1-tDCS led to performance enhancements that were primarily mediated between the daily stimulation sessions, whereas c-tDCS motor skill gains were achieved within the stimulation sessions. This was quantified via a method developed [12,13,14] to mathematically determine the unique contributions of within-session effects (online) and between-session effects (offline) to the overall total motor learning. Taken together, these single- and multi-session c-tDCS studies have important implications for enhancing performance in various motor tasks and in numerous populations.

Despite the promising results in single-day and multi-day c-tDCS studies, all but one of them [11] have involved relatively simple motor tasks such as two-dimensional arm reaching, split-belt walking, eye movements, and unilateral pinch tasks of the hand. In addition, almost all previous c-tDCS studies either involved adaptation-learning paradigms or only a single stimulation session, with the exception of Cantarero et al. (2015) [12]. The motor tasks were also usually laboratory tasks that were novel to the participants and performed in contexts very different from those encountered in everyday life. Therefore, it is unclear at the present time if c-tDCS can improve motor learning in a complex, multi-joint task involving coordination of the whole body with concomitant strict endpoint accuracy requirements. This is an important limitation because while simple motor tasks allow for simpler experiments [17] and more rigorous experimental controls, and facilitate concurrent physiological measurements, the study of complex motor tasks is needed to fully understand movement [18,19]. Complex motor tasks are also more applicable to real world activities performed in settings such as the workplace, military applications, sports, and in activities of daily living.

The primary purpose was to determine the influence of c-tDCS applied over multiple days on motor learning in a complex overhand throwing task in young adults. This was accomplished by requiring two groups of participants to complete three practice sessions on consecutive days concurrent with either tDCS or SHAM stimulation. Based on a single-day c-tDCS overhand throwing study performed in our laboratory as well as a multi-day c-tDCS study [12] and several previous multi-day M1-tDCS studies involving simple motor tasks, it was hypothesized that c-tDCS would enhance overhand throwing accuracy. Accordingly, it was predicted that c-tDCS would lead to greater improvements in online learning, offline learning, and total motor learning compared to practice alone (SHAM stimulation) over 3 days of practice. The secondary purpose was to determine if c-tDCS could increase M1 excitability and whether any potential increases in M1 excitability would be positively correlated with the amount of motor learning induced by c-tDCS. Although previous studies have been mixed regarding the ability of c-tDCS to increase M1 excitability [20], it was hypothesized that if an enhancement of motor evoked potential (MEP) amplitudes obtained from M1 would be observed, the increase would be positively correlated with the magnitude of motor learning displayed by the participants in the c-tDCS group.

A three-dimensional overhand ball throwing task (similar to a baseball throw) was selected as the motor task due to the involvement of the cerebellum in several specific interrelated features of the movement: (1) unconstrained, multi-joint skill that involves the regulation of joint interaction torques [21,22,23,24,25]; (2) the modulation of the finger forces to precisely time the opening of the fingers on a timescale of a few milliseconds [26,27,28,29,30]; (3) the timing and coordination of agonist and antagonist muscle activations [21,28]; and (4) the detection and gradual correction of errors across multiple trials of goal-directed movements [31].

## 2. Materials and Methods

### 2.1. Participants

Thirty young adults participated in the study (16 males and 14 females; mean age: 24.7 ± 3.1; range: 20–31 years; 8 men and 7 women in each group). All participants threw with their right arm and were strongly right-handed based on the Edinburgh Handedness Inventory [32] laterality quotient values. Participants were free of any neurological or psychiatric disorder, had no uncontrolled medical conditions, and did not meet international non-invasive brain stimulation exclusion criteria [33]. In addition, participants were excluded from participating in the study if they were currently competing in a throwing sport at the recreational, collegiate, or professional level. Subjects provided written, informed consent before participating in the study. The experimental procedures were conducted in accordance with the Declaration of Helsinki and were approved by the Institutional Review Board at the University of Nevada, Las Vegas.

### 2.2. Experimental Design

**Figure 1:**
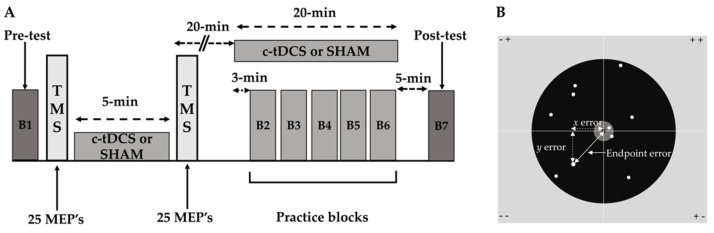
Schematic representation of the major components of the experimental protocol. (**A**) One of the 3 consecutive identical experimental sessions is depicted for illustration purposes. The experimental protocol comprised a pre-test block of overhand throws, a TMS testing paradigm testing the effects of c-tDCS on M1 excitability, 5 practice blocks of overhand throws performed concurrent with 20 min of c-tDCS or SHAM stimulation, and a post-test block of overhand throws; (**B**) the target and the quantification of endpoint error. The entire target area was 1.27 m in length, 1 m in width, the center of the target was 1.71 m from the floor, and the target circle had a diameter of 1 cm. An example data cloud of the endpoints of the ball is depicted for a block of 10 trials along with the x and y errors for a single trial shown that were used to calculate the trial’s endpoint error.

The study employed a double-blind, SHAM-controlled randomized, between-subjects, experimental design. Participants were assigned to either a c-tDCS or a SHAM stimulation group using Research Randomizer (www.randomizer.org, accessed 8-1-2018) by an investigator who did not participate in data collection. Thus, the SHAM condition served as the control or placebo condition, which was implemented using a standard set of procedures from previous tDCS studies that have been shown to not elicit physiological effects (see Section 2.4). In addition, research using this methodology for SHAM stimulation has found that participants cannot distinguish between the active (c-tDCS) condition and the inactive (SHAM) condition. All participants completed 3 experimental sessions on 3 consecutive days at the same time each day. The experimental sessions were identical except that a familiarization involving a short didactic video and an overhand throwing demonstration by one of the investigators was completed at the beginning of the first experimental session. Each session lasted about 1.5–2 h and the following major experimental steps were performed in the order prescribed: (1) pre-test block of overhand throwing trials without stimulation; (2) TMS testing of c-tDCS effects on M1 excitability that collectively involved a TMS pre-test, 5 min of either c-tDCS or SHAM stimulation, and a TMS post-test; (3) practice blocks of overhand throwing trials performed concurrently with 20 min of either c-tDCS or SHAM stimulation; and (4) post-test block of overhand throwing trials (without stimulation). A schematic of these major experimental steps that comprised the experimental protocol is depicted in Figure 1A, whereas the finer methodological details of each step are provided in the subsequent sections. In all the experimental conditions described below, the investigators who conducted the experiments were blind to the group assignment of the subjects. Accordingly, the investigator who was responsible for operating the c-tDCS device and applying stimulation did not participate in any of the other experimental procedures.

### 2.3. Experimental Procedures

#### 2.3.1. Pre-Test Blocks

A pre-test block consisting of 10 overhand throwing trials was performed without concurrent c-tDCS to determine the baseline performance levels for the two groups on Day 1 before any stimulation was applied. Similarly, the pre-test blocks on Days 2–3 were performed in an identical manner and provided a baseline not influenced by stimulation on those days, but possibly influenced by consolidation effects from the previous day. Ten trials per block were chosen for all pre-test blocks as this number was previously determined to be sufficient [11] for baseline data without eliciting an excessive influence on the overhand throwing performance curves during the subsequent practice blocks. In addition, this allowed the number of trials per block to be the same as in the practice and post-test blocks. Finally, the performance of the pre-test blocks without concurrent c-tDCS allowed for the quantification of the contribution of online and offline learning effects to total motor learning (see Section 2.7 Statistical Analysis).

#### 2.3.2. TMS Testing of c-tDCS Effects on M1 Excitability

Single-pulse TMS was performed with a Magstim 200^2^ connected to a double 70 mm remote control figure-of-eight coil. The coil was orientated tangential to the scalp with the handle pointed backwards and laterally at an angle of 45 degrees from the midline. The coil was positioned by an investigator over the “motor hot spot” of the first dorsal interosseous (FDI) muscle of the left M1 to evoke MEPs in the FDI of the right contralateral hand [34]. The electromygraphic (EMG) activity of the FDI muscle was recorded with surface electrodes that were arranged in a belly tendon montage. EMG signals were acquired and recorded using Cambridge Electronic Design (CED; Cambridge, UK) hardware (1902 amplifiers, micro 1401 data acquisition interface) and software (Signal 5.04). All MEPs were evoked at rest while subjects sat in a chair with the forearm on a table, the wrist in neutral with the hand prone, the elbow flexed to ~90 degrees, and the shoulder abducted to ~45 degrees. The subjects were provided FDI EMG feedback on a computer screen and continually monitored by one investigator to assure the FDI was at rest during all recordings.

The TMS aspect of the study proceeded in the following steps: (1) FDI hot spot identification. Participants received suprathreshold TMS pulses as the coil position was optimized so that the scalp area that corresponded to the FDI motor hot spot could be identified; this coil position was marked on a scalp cap, and the scalp cap position on the head was outlined with a mark on the forehead. (2) 1 mV MEP determination. Suprathreshold TMS pulses starting at ~55% of maximum stimulator output (MSO) were applied and the stimulation intensity adjusted while MEPs were monitored and quantified online until the MEPs evoked were as close as possible to a 1 mV peak-to-peak amplitude on average. The software program was then reset to collect the pre-test TMS block. (3) Pre-test TMS block. A total of 25 MEPs were collected using the previously established 1 mV stimulation intensity. (4) Five minutes of c-tDCS or SHAM stimulation. After the previous step, the TMS cap was taken off, the c-tDCS electrode montage was placed on the head, and 5 min of c-tDCS or SHAM stimulation was applied. (5) Post-test TMS block. Immediately after the stimulation time was completed in the previous step, readied investigators acted in coordination as quickly and as accurately as possible to remove the c-tDCS montage, reposition the scalp cap and TMS coil arrangement, and start collection of the post-test TMS block (25 MEPs) immediately using the same 1 mV stimulation intensity as before. During this time, the subject was instructed to remain still and to continue relaxing the hand. (6) Twenty-minute inter-stimulation period. A 20 min time clock was set by one investigator (at the end of the fourth step) who enforced a 20 min time delay between the end of the 5 min c-tDCS application and the subsequent start of the 20 min c-tDCS period associated with the overhand throwing practice blocks (see Figure 1 and below).

This rather complicated and novel paradigm involving a 5 min c-tDCS application followed by a 20 min break was developed to address methodological issues related to MEP measurement before and after tDCS. It was based on research findings by other research groups (described below) in studies that were entirely focused on the influence of different tDCS duration protocols on M1 excitability. The paradigm developed for our study purposes was then extensively piloted in our laboratory for the current study and an identical study that used M1-tDCS (manuscript in press) as opposed to c-tDCS. This was done to assure as much as possible that the paradigm worked as originally intended.

Accordingly, the paradigm was developed relative to three interrelated methodological considerations based on the following: (1) tDCS applied for 3–5 min increase MEPs for 3–5 min after stimulation ends [35,36,37]; (2) if a 20–30 min break is employed before a second tDCS application, the same pattern of MEP increases is observed, whereas inhibition occurs if the break is only 3–10 min [35,36]; and (3) tDCS-induced MEP increases can be obliterated after muscle contractions (task performance), the subject moving, e.g., walking, and other related activities [38,39,40,41], which may render MEP measurement after practice meaningless (for a review of these issues, see Horvath et al. (2014) [39]). Therefore, the paradigm was designed to overcome this limitation while keeping the ability to measure the possible correlations between the increases and the degree of motor learning [42,43,44], but assumes that the second application of tDCS had the same M1 excitability effects as the first [35,36].

#### 2.3.3. Practice Blocks

The practice blocks were performed concurrent with either c-tDCS or SHAM stimulation for a total practice and stimulation period of 20 min (Figure 1A). The practice blocks aspect of the study proceeded in the following steps: (1) the stimulator was turned on for 3 min while subjects stood quietly before performing the first block of overhand throwing trials [11]; (2) a total of 5 blocks of overhand throwing trials were performed with each block comprising 10 overhand throws. These blocks were completed within the remaining 17 min of stimulation as each block took ~1 to 1.5 min to perform and a 2 min rest interval was employed between blocks; (3) the stimulator was kept on after the last block of overhand throws was completed, which was usually 1–2 min to complete the 20 min stimulation period.

#### 2.3.4. Post-Test Blocks

After the practice blocks and the 20 min stimulation period ended, participants stood in place quietly while the inert electrode montage remained on the head, and observed a 5 min rest period before performing the post-test block of 10 trials. The performance of the post-test blocks without concurrent c-tDCS allowed for the quantification of the contribution of online and offline learning effects to total motor learning when incorporated into calculations involving the pre-tests that were performed without stimulation (see Section 2.7 Statistical Analysis).

### 2.4. c-tDCS

A NeuroConn DC Stimulator Plus/MR was utilized to deliver anodal c-tDCS at a current strength of 2 mA via a pair of 5 × 5 cm rubber electrodes that were enclosed in saline soaked sponges. Accordingly, the anode was placed 3 cm lateral to the inion over the right cerebellum (ipsilateral to the right arm), whereas the cathode was placed over the ipsilateral buccinator muscle. The anode and cathode were held in place by separate rubber elastic straps. As mentioned previously, c-tDCS was applied for 5 min between the TMS pre-test and post-test blocks and for 20 min during the practice blocks of overhand throws using the same stimulation parameters. During the overhand throwing trials, the stimulation device was placed in the small backpack [11], whereas the stimulator was placed behind the participant on a table in association with MEP testing protocol. Although other c-tDCS parameters are possible and some have yielded positive effects [5], the aforementioned combination set of c-tDCS polarity, montage, current strength, and duration was chosen as it had the most previous studies that have demonstrated positive effects [6,7,8,9,10,12]. Most importantly, this included our previous single-session overhand throwing c-tDCS study conducted in the same laboratory [11]. SHAM stimulation was applied according to standard procedures [45,46]. Accordingly, current was ramped up to over 10 s, held constant at 2 mA for 30 s, and ramped back down over 10 s, which has been shown to induce the same scalp skin sensations as real c-tDCS without exerting any physiological effects.

### 2.5. Overhand Throwing Task

The overhand throwing task was identical to a previous study [11] and performed using very similar experimental procedures. Participants stood behind a line on the floor located at a distance of 6 m from a cement wall. A wooden board was tightly screwed into the wall and a laminated poster that was further encased in clear tape was mounted on the board. The poster depicted a large target area with a very small (1 cm diameter) “bull’s-eye” center (Figure 1B).

Participants threw a tennis ball with their dominant right arm in a manner consistent with a baseball throw and were instructed to execute each throw as accurately as possible by attempting to hit the center of the target. Participants used their visual feedback of the ball’s endpoint relative to the center of the target after each trial and were told to use that information to minimize the error distance between the ball’s endpoint and the target center on subsequent trials. An investigator who stood near the participant covered the ball with red chalk before and midway through each block of 10 trials so that marks were made denoting final endpoint position of the ball upon hitting the target area. The same investigator retrieved the ball after it had rebounded back off of the target area on the wall and handed it to the participant after each trial. Each mark was recorded with a small trial-numbered circular sticker after each trial by a second investigator who stood near the target area. After each trial block (participants’ inter-block rest interval), the sticker endpoint x, y coordinates were measured, recorded, and entered directly into a file on a laptop computer by 2–3 investigators. Finally, the stickers were removed from the target area between trial blocks and the process repeated for the next trial block.

The overhand throwing task was executed identically in all trial blocks and always conducted while wearing a small, tightly fitting backpack with the tDCS device placed inside. Importantly, the tDCS device was only turned on during the practice blocks (Figure 1), but was not on during the test-blocks though the inert electrode montage remained on the head of the participant. The configuration of the backpack, stimulator, and associated tDCS electrode montage did not restrict task performance [11]. Thus, overhand throws were always conducted in the same experimental conditions and in an unconstrained manner in 3-dimensional space. Taken together, the overhand throwing task, small target size, and long throwing distance were all task details that were specifically selected within the laboratory space limits to assure that the motor task would represent a very difficult motor skill.

### 2.6. Data Analysis

The primary dependent measure of interest was the endpoint error, whereas the secondary dependent measure of interest was the MEP amplitude obtained from TMS applied to M1. The dependent measures of age, laterality quotient, and 1 mV MEP intensity were also quantified and viewed as control measures. The endpoint error was quantified in the same manner as in previous studies [11,47,48,49]. The Pythagorean Theorem was utilized to determine the shortest absolute distance between the x, y coordinates of the target center and the final endpoint x, y coordinates of the ball (Figure 1B). For a detailed description of endpoint error calculation in goal-directed tasks, see Poston et al. (2013) [50]. The ball’s endpoint coordinates were entered into a custom-written program in Microsoft Excel, which calculated the endpoint error for each trial. The average endpoint error of the 10 overhand throwing trials performed in each trial block was taken as the final endpoint error value for analysis. MEP data were analyzed offline using a customized script written in Signal software (Cambridge Electronic Design, Cambridge, UK). The MEP size was calculated as the peak-to-peak amplitude for each MEP and the average of the 25 MEPS in each TMS test block was taken for analysis. For the control measures, the average age and laterality quotient was calculated for each group, whereas the average 1 mV MEP intensity for each subject across the 3 days was calculated and then these values were averaged for the two groups.

### 2.7. Statistical Analysis

Endpoint error was analyzed using a methodology that was mainly similar to the previous 3-day c-tDCS study by Cantarero et al. (2015) [12], but also shared some features similar to our previous single- and multiple-day studies [11,47,48]. The endpoint error analysis proceeded in three steps: (1) endpoint error obtained from only the test blocks was analyzed with a 2 Group (c-tDCS, SHAM) × 3 Day (1, 2, 3) × 2 Test (pre-test, post-test) three-way mixed ANOVA. This analysis was conducted using only endpoint error data from the test blocks as stimulation was not applied during these blocks. This also allowed for the results to be able to be compared to the results of Cantarero et al. (2015) [12]; (2) each endpoint error from each day (test blocks and practice blocks) was analyzed with a two-way mixed ANOVA: 2 Group (c-tDCS, SHAM) × 3 Day (1, 2, 3). Thus, this second analysis used the average endpoint error value of all 7 blocks combined (2 test and 5 practice blocks) performed for each day. This was done to complement the first analysis because pilot data, a previous single-day study [11], and the current study all had many individual participant instances where performance in the test block could differ rather substantially from some of the practice blocks. This was almost certainly due to the difficulty of this motor task. Thus, this analysis could, at least potentially, better represent the overall average performance for each day; and (3) the online, offline, and total learning effects were compared between the two groups using unpaired two-tailed *t*-tests.

The MEP amplitude data were analyzed with a three-way mixed ANOVA: 2 Group (c-tDCS, SHAM) × 3 Day (1, 2, 3) × 2 Test (pre-test, post-test). In addition, bivariate linear regression analyses were used to examine the association between the change in MEP amplitudes between the TMS pre-tests and post-tests and the change in endpoint error (endpoint accuracy) between the pre-test and post-test blocks for the two groups. These correlations were repeated for each of the days. For the control measures, the age, laterality quotient, and 1 mV MEP intensity differences between groups were analyzed with separate unpaired two-tailed *t*-tests. For all the ANOVAs described above, post hoc comparisons using Bonferroni adjustment for multiple comparisons were performed when appropriate to locate where significant differences occurred between pairs of means. The significance level was set at α < 0.05 for all above analyses and data are depicted as means ± standard errors in the figures.

## 3. Results

### 3.1. Endpoint Error

**Figure 2:**
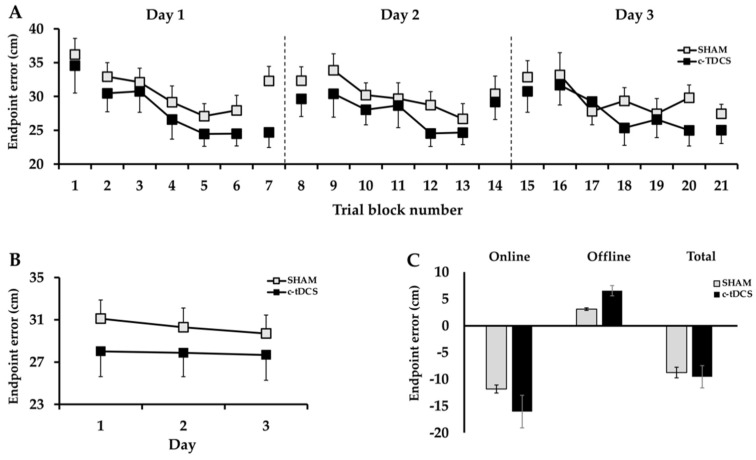
Endpoint error in the overhand throwing task averaged over all 7 daily trial blocks for the c-tDCS and SHAM groups. (**A**) Endpoint error declined across the test blocks for the 3 days of practice (*p* = 0.02), but the decline was similar for the c-tDCS and SHAM groups (*p* = 0.332); (**B**) endpoint error was similar for the two groups (*p* = 0.381) and across the 3 days (*p* = 0.507); (**C**) the online (*p* = 0.325), offline (*p* = 0.188), and total learning (*p* = 0.843) were similar for the c-tDCS and the SHAM groups.

Motor learning differences between groups were compared across practice days and test blocks with a 2 Group (c-tDCS, SHAM) × 3 Day (1, 2, 3) × 2 Test (pre-test, post-test) ANOVA and are depicted in Figure 2A. There was a significant Day × Test interaction (*p* = 0.050, *η*^2^ = 0.102) and post hoc analyses of the interaction indicated that endpoint error when collapsed across Group was significantly lower in the post-test compared to the pre-test on Day 1 (*p* < 0.001) and Day 3 (*p* = 0.002), but not Day 2 (*p* = 0.491). There was also a significant main effect for Day (*p* = 0.02, *η*^2^ = 0.131) and post hoc analysis indicated that endpoint error when collapsed across Group and Test was lower on Day 3 compared to Day 1 (*p* = 0.048). However, the pairwise mean comparison between Day 2 and Day 3 along with the pairwise mean comparison between Day 1 and Day 2 were both non-statistically significant (*p* = 0.433 and *p* = 0.35, respectively). There was also a significant main effect for Test (*p* < 0.001, *η*^2^ = 0.45), which indicated that endpoint error was lower in the post-tests compared to the pre-tests. The main effects for Group (*p* = 0.332, *η*^2^ = 0.034), Group × Test interaction (*p* = 0.404, *η*^2^ = 0.025), Group × Day interaction (*p* = 0.359, *η*^2^ = 0.036), and Group × Day × Test interaction (*p* = 0.268, *η*^2^ = 0.046) were all non-statistically significant.

Motor learning differences between groups were also compared across practice days using average endpoint error data collapsed across all the practice and test blocks with a 2 Group (c-tDCS, SHAM) × 3 Day (1, 2, 3) mixed ANOVA. The analysis revealed that the Group × Day interaction (*p* = 0.773, *η*^2^ = 0.009; Figure 2B), the main effect for Day (*p* = 0.507, *η*^2^ = 0.024), and the main effect for Group (*p* = 0.381, *η*^2^ = 0.028) were all non-statistically significant.

To determine differences between groups in online, offline, and total motor learning, a series of separate unpaired two-tailed *t*-tests were used to compare online, offline, and total learning effects between groups. The analyses revealed that the online (*p* = 0.404), offline (*p* = 0.353), and total learning effect (*p* = 0.818) were all non-statistically significant between the c-tDCS and SHAM groups (Figure 2C).

### 3.2. MEP Amplitude

**Figure 3:**
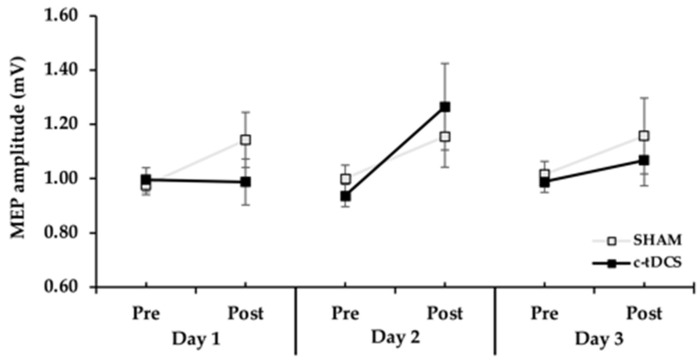
MEP amplitude in the TMS pre-tests and post-tests for the 3 days in the c-tDCS and SHAM groups. MEP amplitude was significantly increased between the pre-test and post-test on all 3 days (Test main effect, *p* < 0.011), but the increase was not statistically significant between the c-tDCS and the SHAM groups (*p* = 0.677).

MEP amplitude differences were compared between groups across practice days and test blocks with a 2 Group (tDCS, SHAM) × 3 Day (1, 2, 3) × 2 Test (pre-test, post-test) ANOVA. There was a significant main effect for Test (*p* = 0.011, *η*^2^ = 0.211, Figure 3), which indicated that when collapsed across group MEP amplitude was higher in the post-tests compared to the pre-tests. However, the main effect for Group (*p* = 0.677, *η*^2^ = 0.006), main effect for Day (*p* = 0.479, *η*^2^ = 0.026), Group × *Test* interaction (*p* = 0.835, *η*^2^ = 0.002), Group × Day interaction (*p* = 0.629, *η*^2^ = 0.016), Test × Day interaction (*p* = 0.213, *η*^2^ = 0.054), and Group × Day × Test interaction (*p* = 0.192, *η*^2^ = 0.057) were all non-statistically significant.

### 3.3. Associations between Increases in MEPs and Increases in Endpoint Accuracy

**Figure 4:**
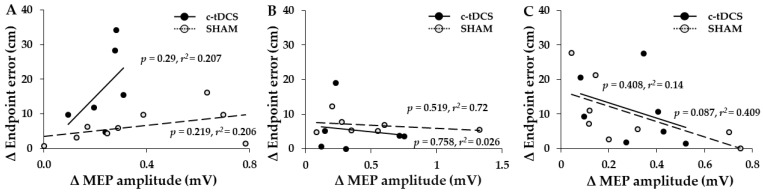
Associations between increases in MEP amplitude and increases in endpoint accuracy. (**A**–**C**) The absolute change (increase) in endpoint accuracy (decrease in endpoint error) was not associated with the absolute change (increase) in MEP amplitude for the participants in either group that displayed both increases in endpoint accuracy and MEP amplitude.

Separate bivariate linear regressions were performed for each day and only using participants who displayed an increase in both MEP amplitude and endpoint accuracy were included in the analyses. The analyses revealed that the associations between the change in MEP amplitudes between the TMS pre-tests and post-tests and the change in endpoint error (endpoint accuracy) between the pre-test and post-test blocks for the two groups were all non-statistically significant (*p* value range: 0.087–0.758) and characterized by very low *r*^2^ values (range: 0.026–0.72) as indicated in Figure 4A–C.

### 3.4. Control Measures

Separate unpaired *t*-tests revealed that differences between groups for age (*p* = 1.00), laterality quotient (*p* = 0.602), and the 1 mV MEP intensity (*p* = 0.754) were all non-statistically significant.

## 4. Discussion

The primary purpose was to determine the influence of c-tDCS applied over multiple days on motor learning in a complex overhand throwing task in young adults. The secondary purpose was to determine if c-tDCS could increase M1 excitability and if any potential increases in M1 excitability would be positively correlated with the amount of motor learning induced by c-tDCS. There were four main findings: (1) overhand throwing accuracy improved over the 3 days of practice, but the magnitude of reduction in endpoint error achieved at the end of practice was not significantly different between the c-tDCS and SHAM stimulation groups; (2) the relative influences of online and offline learning on the total motor learning were also similar between the two groups; (3) M1 excitability was increased for both the c-tDCS and SHAM groups, but the increases in M1 excitability were similar for the two groups; and (4) increases in endpoint accuracy were not associated with increases in MEP amplitude even when comparisons were restricted to participants in either group that displayed both increases in endpoint accuracy and MEP amplitude. Collectively, these results indicate that three consecutive daily applications of c-tDCS does not improve motor learning in a very complex motor task in young adults or significantly increase M1 excitability to a greater degree than practice alone.

### 4.1. Effects of c-tDCS on Motor Learning

Motor learning is defined as a relatively permanent improvement in motor performance due to practice. The physiological mechanisms and adaptations underlying the motor learning process are complex and occur in numerous brain regions [51,52], over different time scales [51,53], and vary depending on the details of the motor task [54]. Nonetheless, classic research over many years has shown that M1 and the cerebellum are brain areas that play the predominate roles in motor skill learning [52,54]. Accordingly, this is one major reason that non-invasive brain stimulation methods such as tDCS have targeted these brain areas the most frequently when attempting to enhance motor performance [1,2,4]. However, the vast majority of these studies have investigated relatively simple motor tasks (see tables in these reviews [1,2]) that were rather novel to the participants.

The present study was the first to investigate the influence of c-tDCS on motor learning over multiple days in a complex motor task involving whole body coordination with strict endpoint accuracy requirements. The original hypotheses were that the c-tDCS group would exhibit significantly greater motor learning at the end of the 3 days of practice compared to the SHAM group. Furthermore, it was expected that most of the improvements in total motor learning in the c-tDCS group would be realized through online effects while offline effects would play a much smaller role. Contrary to this set of predictions, the reductions in endpoint error across the three practice sessions were nearly identical for the c-tDCS and SHAM groups. In fact, all aspects of the entire performance curve were comparable as the between-group differences were only 1.6 cm in the pre-test on Day 1 (baseline), modulated similarly across the 3 days, and only 2.4 cm difference in the post-test on Day 3 (Figure 2A). Accordingly, there were also no differences between groups in the relative contributions of online and offline learning to the total motor learning (Figure 2C).

The findings of the current study are not consistent with the findings of the majority of the initial previous single-session c-tDCS studies by other research groups [6,7,8,9,10], although most of these studies used adaptation-learning paradigms. Furthermore, the findings differ from a very recent study that reported that c-tDCS improved strength and coordination in full-body motor tasks in gymnasts [55]. However, this study used a novel bilateral electrode montage. Most notably, the results are also in contrast to an earlier study performed in our lab [11] that used the same overhand throwing task, the same c-tDCS parameters, and a very similar experimental paradigm, except the prior study only had one day of c-tDCS application. In that study, the decline in endpoint error was greater for the c-tDCS group compared with the SHAM group at the end of the practice session and this difference was maintained in a retention test completed a day later (no stimulation on the retention day). The present outcomes are also in contrast to the one available 3-day c-tDCS and motor skill study [12], where extremely large enhancements in motor skill were observed for the c-tDCS group compared to the SHAM group in the SVIPT. This is the most comparable study as we intentionally chose to have three practices sessions, use the same c-tDCS parameters, and employ similar statistical analysis, but with an overhand throwing task as opposed to the SVIPT. This was also done to simultaneously try to extend our previous single-session overhand throwing c-tDCS study [11]. Other than the obvious possible differences due to the motor task utilized, the reasons for these disparate findings between the two studies are not clear.

However, the present results are similar to a series of more recent studies performed in a range of contexts, which have found little to no positive effects of c-tDCS on motor performance [3]. Interestingly, two separate research groups each failed to replicate a previous c-tDCS study performed either in the same lab [56,57] or by some of the same researchers [8,58]. This was despite the motor tasks being quite different to each other as one set of studies involved conditioned eyeblink responses and the other set involved arm reaching movements with a pen held in the hand. Similarly, the current study also failed to replicate most, but not all, aspects of our prior single-session c-tDCS study using the same motor task. As mentioned before, that study showed improved throwing scores at the end of practice on Day 1 and in a retention test the next day. Thus, the overall results of the current study and that previous study are not compatible. However, in the current study, the endpoint error on Day 1 was substantially lower in the post-test block in the c-tDCS group just as in Day 1 of the previous study. However, the lack of a Group × Day interaction precluded this from being evaluated statistically in the current study. Furthermore, although comparisons of those two data points look similar to the previous study, a close examination of the performance curves reveals other differences. For example, the current study had neither adjacent practice blocks before the post-test on Day 1 nor the pre-test on Day 2 with endpoint errors that were substantially lower for the c-tDCS group, as in the previous study. Thus, it is very difficult to know if the post-test performance on Day 1 was due to c-tDCS as opposed to random variation in the data. Thus, it cannot be ruled out completely that c-tDCS had a small, but non-statistically significant effect of slightly accelerating the rate of motor learning on Day 1. Nonetheless, any possible advantage of c-tDCS by the end of Day 1 was transient and not evident on Days 2 and 3. Therefore, it appears that the current results represent a third set of c-tDCS studies in the literature performed by the same research groups that could not replicate their own previous results. In addition, other recent studies have reported that c-tDCS failed to enhance performance in a whole-body balance task [59] and an adaptation task involving moving a joystick with the hand and wrist [60]. Taken together, all these results provide support for the current findings and strongly suggest that c-tDCS effects on motor performance may not be as strong or consistent as initial studies indicated.

### 4.2. Effects of c-tDCS on M1 Excitability

The application of anodal tDCS to M1 usually results in both increases in motor skill and increases in M1 excitability as measured via MEPS evoked by TMS. Furthermore, these increases in motor skill and MEPs were positively correlated in some initial studies [43,44]. Therefore, it was initially assumed that the increases in M1 excitability were at least partially responsible for the improvements in motor skill. Accordingly, a handful of c-tDCS studies have measured changes in MEPs obtained from M1 following c-tDCS, ostensibly with the rationale that c-tDCS-mediated increases in M1 excitability could also be a mechanism underlying motor skill improvements with c-tDCS. However, a review and meta-analysis of previous studies on the topic found mixed results. Increases and decreases as well as no changes in M1 excitability were all reported after c-tDCS application [20]. Therefore, the secondary purpose of the current study was to examine if increases in M1 excitability occur with c-tDCS, and if these increases would be positively associated with improvements in endpoint accuracy.

The major finding was that MEP amplitude was significantly increased in both the SHAM and c-tDCS from the pre-tests to the post-tests when the results were averaged across the 3 days (Figure 3). Thus, the significant MEP increase in the SHAM group was unexpected, although small increases in MEP have been observed in many M1-tDCS studies following SHAM stimulation [61]. Thus, this type of result is not a rare occurrence when measuring MEPs in tDCS studies. However, the absolute increases were very small for both groups (13.9–15.5%) and only approximately 40% of subjects in each group displayed an increase in MEPs on a given day. This magnitude of increase is well below the range of MEP increases (~20–50%) typically observed in M1-tDCS studies [39,61,62]. In addition, another recent study in our lab (manuscript in press) used the exact same experimental paradigm, with the exception that M1-tDCS was used. This study found significant MEP increases of 47% in the M1-tDCS group and only a non-significant 5% increase in the SHAM group. Based on these collective lines of reasoning, the daily increases in MEP in the SHAM group were most likely due to the large inherent variability involved in MEP measurements and random variation in the MEP data [61], which are issues that have been described and analyzed extensively (see [39,62] for reviews). However, the possible contributions of small c-tDCS or placebo effects cannot be completely ruled out. Nonetheless, the lack of significant positive associations in MEP amplitude changes and endpoint accuracy changes and very small *r*^2^ values (Figure 4A–C) indicate that the MEP increases likely had little functional significance relevant to motor learning. The absence of significant positive associations between changes in MEPs and changes in endpoint accuracy is consistent with a previous comprehensive study, which found that MEP increases elicited by M1-tDCS were not associated with the amount of motor learning achieved by subjects across a range of motor tasks [42].

### 4.3. Possible Reasons for the Failure of c-tDCS to Improve Overhand Throwing Accuracy

The lack of statistically significant results of the current study, the failure of c-tDCS replication studies, and other recent negative studies suggest that it should not be presumed that application of c-tDCS almost always elicits improvements in motor skill in young adults. Nonetheless, there are a few possible factors that could have been responsible for the lack of an ability of c-tDCS to enhance motor learning in the present study. First, it could be argued that the c-tDCS parameters were suboptimal. This view is supported by the fact that various combinations of electrode montage, polarity, current strength, timing relative to task performance, and stimulation duration have also shown efficacy [5]. Although the issue of other optimal stimulation parameters cannot be ruled out, this possibility is unlikely as the current set of c-tDCS parameters was selected specifically because it had the highest number of total positive study outcomes relative to other sets of parameters [6,7,8,9,10,11,12]. Most importantly, the identical parameters were successful in improving the same overhand throwing task in our laboratory even though this study involved only a one-time c-tDCS application [11]. Second, it is conceivable the group of participants randomly assigned to the c-tDCS group may have contained a relatively high number of non-responders as some studies have shown that a moderate number of people may be non-responders to M1-tDCS [63]. However, it should be pointed out that these studies defined non-responders based solely on TMS cortical excitability measures taken from M1 (resting motor threshold, 1 mV MEP) in response to tDCS. Thus, they did not measure motor performance at all. Accordingly, the most comprehensive study on the topic found that MEP increases elicited by M1-tDCS (and two other forms of non-invasive brain stimulation) were not associated with the amount of motor learning achieved by subjects in several motor tasks [42]. Thus, no direct studies have been conducted in an attempt to discriminate between responders and non-responders to c-tDCS based on a combination of TMS and motor learning outcomes, which renders this explanation plausible, but extremely speculative. Nevertheless, there could be variations across individuals in the amount of current delivered to cerebellar neurons due to dissimilarities in many physiological, biological, and anatomical factors. For example, differences in the nerve fiber orientation are thought to be one major factor responsible for the effective amount of current reaching cerebellar neurons [3,64]. These possibilities will have to be examined in subsequent studies that combine behavioral and several physiological measures. Finally, a combination of the above factors could be responsible for the lack of c-tDCS effects on motor learning in the current study.

Although the aforementioned factors could potentially have contributed to the current findings, other possible factors such as the baseline skill level, age of the participants, TMS stimulation intensity to evoke a 1 mV MEP [65], and degree of right-handedness do not apply to the current findings, as these factors were almost exactly the same between groups. Furthermore, other common criticisms of tDCS studies that observe negative effects, such as only one day of stimulation or the use of a motor task that is not amenable to tDCS, are also not relevant. This is because the current study involved 3 days of stimulation using the same motor task that was improved with c-tDCS in our single-day study [11]. Collectively, these lines of reasoning imply that the present study design should have been able to find performance enhancements induced by c-tDCS if they existed.

### 4.4. Limitations and Future Directions

Although the findings were clear in regard to the absence of positive effects of c-tDCS on motor learning, the study had several limitations that should be addressed in future research. These are mainly related to the interrelated issues of choice of stimulation parameters, the population studied, and individual differences in response to c-tDCS. The parameters of c-tDCS (e.g., electrode montage, current strength) were chosen because they had increased motor skill in the greatest number of previous studies by another research group [6,7,8,9,10,12] and in our previous single-session study [11]. Nonetheless, it is possible that another set of c-tDCS parameters could be more effective. For example, a recent study used bilateral anodal c-tDCS and demonstrated improvements in complex whole-body tasks in gymnasts [55]. Another study found that a bilateral electrode montage (anode over the right cerebellar hemisphere, cathode over the left cerebellar hemisphere) and a 4 mA current strength outperformed several other unilateral montage and lower-current-strength combinations. However, this study involved gait and balance performance in Parkinson’s disease patients. Nevertheless, it is possible that bilateral montages or increasing the total dose of c-tDCS through greater current strengths could be superior to the stimulation parameter used here. Other related types of non-invasive brain stimulation such as transcranial alternating current (tACS) applied to cerebellum alone [66,67] or to cerebellum and M1 simultaneously [68,69,70] have also elicited significant improvements in motor skill in healthy adults. Therefore, future research is warranted to further examine these other sets of cerebellar-stimulation parameters.

The other notable limitations of the study were that the application of c-tDCS was not individualized for each participant and the only population studied were healthy young adults in a tight age range. Recently, initial research has attempted to optimize cerebellar-stimulation parameters based on participant anatomy [71] by using neuronavigation to focus stimulation on cerebellar lobule VIII in an individualized manner. This strategy along with modulating current strength could be particularly important when applying c-tDCS to healthy young adults or children with movement impairments that involve cerebellar contributions [72,73,74] compared with healthy [75] or diseased older populations with cerebellar dysfunction [76]. This is because the cerebellum cortex is highly convoluted in nature, has a high variation in nerve fiber orientation [3], and shrinks with advanced age [77], which strongly implies that individualization of cerebellar stimulation could further enhance performance outcomes. In summary, further research could explore the synergies between unilateral and bilateral electrode montages as well as varying c-tDCS dose parameters such as current strength, duration, and days of stimulation. Based on the available research, these strategies have significant potential to enhance motor skill acquisition and motor learning across different populations and motor tasks.

## 5. Conclusions

Participants were able to progressively decrease endpoint error across the three consecutive days of practice, but these improvements in endpoint accuracy were similar between the c-tDCS and SHAM stimulation groups. Therefore, c-tDCS failed to improve motor learning in this complex motor task to a greater degree than practice alone in the current experimental conditions. In addition, c-tDCS did not significantly increase M1 excitability to a greater extent than SHAM stimulation. Furthermore, when increases in M1 excitability occurred, they were not positively associated with improvements in endpoint accuracy for either group. When these results are considered in the context of the overall c-tDCS and motor skill literature, they are consistent with recent replication studies [56,57] that have suggested that the effects of c-tDCS may not be as robust as initial studies indicated [8,58]. Therefore, future studies are needed to fully determine the efficacy of c-tDCS for potentially enhancing motor skill acquisition and learning in healthy young adults. Finally, interindividual differences in the motor performance responses elicited by c-tDCS and the physiological mechanisms underlying these will be especially important, but challenging issues that should be addressed in future work.

## Data Availability

The data presented in the study are available on request from the corresponding author.
